# Assessing the performance of bloodfed mosquito collection strategies in Australia

**DOI:** 10.1093/jme/tjaf139

**Published:** 2025-10-16

**Authors:** Kevin Thabo Moore, Eloise Skinner, Patrick Norman, Kirsty Richards, Geoff Pollock, Hamish McCallum, Brendan Trewin

**Affiliations:** School of Environment and Science, Griffith University, Nathan, QLD, Australia; UQ Centre for Clinical Research, Faculty of Health, Medicine and Behavioural Sciences, The University of Queensland, Brisbane, QLD, Australia; Climate Action Beacon, Griffith University, Southport, QLD, Australia; SunPork Group, Brisbane, QLD, Australia; SunPork Group, Brisbane, QLD, Australia; School of Environment and Science, Griffith University, Nathan, QLD, Australia; CSIRO Health and Biosecurity, Dutton Park, QLD, Australia

**Keywords:** feeding behavior, vector ecology, surveillance, mosquito borne disease

## Abstract

Bloodfed mosquitoes provide direct evidence of host use, a critical factor for understanding and managing mosquito-borne diseases, yet they are difficult to collect. This study assessed 6 bloodfed mosquito collection methods: direct aspiration, 2 commercially available surveillance traps, and 3 types of artificial resting shelters (ARS) at commercial piggeries and in nearby wetlands within Australia. Methods were evaluated for collection success (ability to capture bloodfed mosquitoes in field conditions) and efficiency (bloodfed mosquitoes collected per minute of effort), while also evaluating the influence of temperature, precipitation, and water index on trapping outcomes using a generalized linear mixed model. Of the 2,530 bloodfed mosquitoes collected, aspiration achieved the highest overall success, largely due to 2 exceptionally productive sites. Two ARS (large bins and felt bags), however, performed more consistently across locations and were particularly efficient in locations with fewer bloodfed mosquitoes. Collection method strongly influenced species composition: aspiration and ground-level ARS collected primarily *Culex annulirostris* Skuse (Diptera: Culicidae), whereas felt-bag ARS suspended from trees captured proportionally more *Anopheles annulipes* Walker (Diptera: Culicidae). Aspiration and ARS were most effective near dense, humid ground vegetation: habitats that concentrate resting mosquitoes. These findings suggest a 2-step approach to optimize collection outcomes. First, identify natural resting locations, then match the collection to local abundance and target species: aspiration for ground vegetation with abundant *Culex* species, ARS (especially felt bags) for low-abundance or arboreal-resting species. This framework supports more targeted and efficient surveillance of mosquito feeding patterns.

## Introduction

Mosquito-borne viruses are a persistent and evolving threat to human, livestock, and wildlife health worldwide (Franklinos et al. 2019). Transmission is difficult to stop because many of these pathogens spread between multiple hosts and vectors in complex networks (Kilpatrick and Randolph 2012). Bloodfed mosquitoes are a valuable resource for understanding mosquito feeding patterns and the links between hosts and vectors that underpin pathogen transmission (Fikrig and Harrington 2021). They can reveal host use, identify potential bridge vectors, and even be used in xenosurveillance to detect pathogens and antibodies in host blood (Vieira et al. 2024). Collecting bloodfed mosquitoes, however, is notoriously difficult, and methods for doing so remain poorly standardized (Melgarejo-Colmenares et al. 2022). This study addresses that gap by evaluating collection techniques in Australian agricultural settings, providing the methodological foundation for future research on mosquito–host associations and virus ecology.

Collecting bloodfed mosquitoes is difficult because their behavior changes after feeding (Melgarejo-Colmenares et al. 2022). Once engorged, female mosquitoes abandon host-seeking behavior and retreat for several days to resting sites that offer protection from desiccation and predation (Silver 2008). These resting sites differ among species, with preferences ranging from dense vegetation and animal shelters to artificial containers and structures (Edman et al. 1968, Burkett-Cadena et al. 2008, 2013). This shift in behavior renders conventional traps baited with host cues, such as carbon dioxide (CO_2_)-baited light traps, largely ineffective, as they primarily attract unfed mosquitoes in search of a bloodmeal (Silver 2008). When bloodfed mosquitoes are captured by these traps they are usually partially engorged due to interrupted feeding, which can potentially bias host-feeding studies toward defensive hosts (Thiemann and Reisen 2012). CO_2_-baited light traps also tend to favor certain mosquito species, limiting their representativeness (Giordano et al. 2020). Further, the need for a continuous CO_2_ supply reduces their feasibility for targeted bloodfed ­mosquito surveillance over periods greater than 1 to 2 d (Silver 2008).

To address the limitations of CO_2_-baited light traps, alternative collection methods have been developed that target bloodfed mosquitoes (Silver 2008). Aspiration, one of the most widely used approaches, involves manually collecting mosquitoes from natural resting areas using battery-powered or manual suction devices (Silver 2008). All aspirators function as suction devices with a tube or nozzle that allows the user to draw mosquitoes into a collection chamber, often fitted with a mesh screen to prevent escape (Silver 2008). This method enables sampling across diverse habitats including tree hollows, animal burrows, and rock crevices but is labor-intensive and requires trained operators (Breeland 1972, Service 1977, Burkett-Cadena et al. 2013). Popular alternatives to aspiration are artificial resting shelters (ARS), which mimic natural resting environments (Edman et al. 1968, Burkett-Cadena et al. 2008, Brown et al. 2018, Alemayehu et al. 2020, Jaworski et al. 2022). The most common ARS design is a box with one side removed and placed on the ground. Mosquitoes are usually removed from the ARS with an aspirator, although some “pop up” ARS have clever designs that allow for human-powered collection to great effect (Silver 2008, Burkett-Cadena et al. 2019, Alemayehu et al. 2020). Studies suggest that larger, darker shelters placed at strategic locations improve collection success (Jaworski et al. 2022). In studies that directly compared the success of different bloodfed mosquito collection methods, ARS and aspiration were usually more successful than traditional attractant traps (Friesen and Johnson 2013, Hernandez-Colina et al. 2021). Other collection methods, such as sweep nets, gravid traps, pit traps, truck traps, and Biogents Sentinel traps offer alternate approaches but come with practical constraints (Silver 2008). Selecting an appropriate collection method requires balancing efficiency, feasibility, and the ecological context in which trapping occurs. Likewise, methods differ in the species they capture due to species specific resting preferences (Burkett-Cadena et al. 2013).

In addition to trap design, environmental conditions also shape mosquito behavior and collection outcomes. Temperature, humidity, and vegetation structure can influence where bloodfed mosquitoes rest, while rainfall is important for breeding site availability and seasonal population fluctuations (Sauer et al. 2021). These factors introduce variability across the landscape, making collection efficiency as much a function of habitat conditions as of the method itself (Sauer et al. 2021). To generate meaningful comparisons across collection techniques, it is important to account for the environmental context in which they are deployed.

This study was conducted as part of a broader investigation into Japanese encephalitis virus (JEV) transmission at Australian piggeries, a project that required the rapid collection of bloodfed mosquitoes across multiple sites. The 2021/2022 outbreak of JEV in Australia underscored the importance of understanding mosquito feeding behavior at piggeries, yet at the time of the outbreak little was known about which mosquitoes were feeding on or around domestic pigs, and specifically which species were acting as bridge vectors between wild bird hosts and domestic pigs (Mackenzie et al. 2022, Richards 2023). Likewise, bloodfed mosquitoes had never been collected in these locations. Here we focused only on testing collection methods for bloodfed mosquitoes; future studies will use these methods to examine mosquito feeding patterns at agricultural settings. Our assessment of 6 bloodfed mosquito collection methods focused on their success (the overall ability of a method to collect bloodfed mosquitoes under field conditions), efficiency (bloodfed mosquitoes collected per minute of effort), and response to environmental conditions, to determine which method is most suitable for large-scale, rapid deployment across varied environments in Australia.

## Materials and Methods

### Site Locations

Bloodfed mosquitoes were collected from 6 sites in Queensland and Victoria, Australia, over a total of 24 collection days, from November 2023 to March 2024. The collection sites were within and around 5 commercial piggeries and one grassland pasture. To protect the privacy of the properties, their names and locations are not provided but their general locations and the surrounding environmental landscapes are described below.

Piggery 1 is a large production site in southern Queensland (6 sheds; 3.42 ha of sheds), west of the Great Dividing Range, surrounded by *Acacia* and *Eucalyptus* woodlands, as well as grazing pastures. Piggery 2 is a medium-sized production site in northern Victoria (5 sheds; 0.99 ha of sheds), surrounded by cultivated pastures. Piggery 3, also in northern Victoria, is a medium-sized piggery (5 sheds; 0.76 ha of sheds) located next to a wetland and surrounded by grazing pasture. Piggery 4, in northern Victoria, is a large site (9 sheds; 1.96 ha of sheds) situated near a ridge with a large *Eucalyptus* forest. Cattle 1 is large cattle property in southern Queensland with a mixture of grassland pastures and forests. Collections at this property took place at small ponds in an open pasture near Brigalow forests. Cattle 1 was included in this study due to its high density of feral pigs, a potentially important host for JEV transmission. Piggery 5 is a small family farm in the Atherton Tablelands area of northern Queensland (1 shed; 0.1 ha of sheds), surrounded by grazing pasture.

### Trap Designs

Six collection methods were evaluated: 3 types of ARS (large bins, felt bags, and small bins), Pacific Biologics Light Traps (PB: Pacific BioLogics Pty Ltd, Australia), Biogents Sentinel traps (BG-S: Biogents AG, Regensburg, Germany), and a homemade battery powered aspirator based on the Prokopack aspirator design (Vazquez-Prokopec et al. 2009).

The large bins were chosen for their size, ease of deployment, and dark color. They were black plastic cylinders with a volume of 60 L, measuring 43 cm in diameter and 50 cm in height. Previous research has demonstrated that larger and darker resting boxes are more successful at attracting bloodfed mosquitoes than smaller, lighter ones (Edman et al. 1968). The inside surface of the large bins was sanded with 60 grit sandpaper to help mosquitoes grip the plastic while resting. Before deployment the large bins were placed outside in the sun for 2 d to rid them of volatile organic compounds. Each large bin cost AUD $15. Small bins were selected for their low cost, ease of transport, and compact size; each had a volume of 9.3 L and measured 26 cm in diameter and 25 cm in height. Made of black plastic, they were placed in similar locations as the large bins. Their interior surfaces were sanded with 60 grit sandpaper and, as with the large bins, they were left outside for 2 d to off-gas volatiles. Each small bin cost AUD $4.

**Fig. 1.**
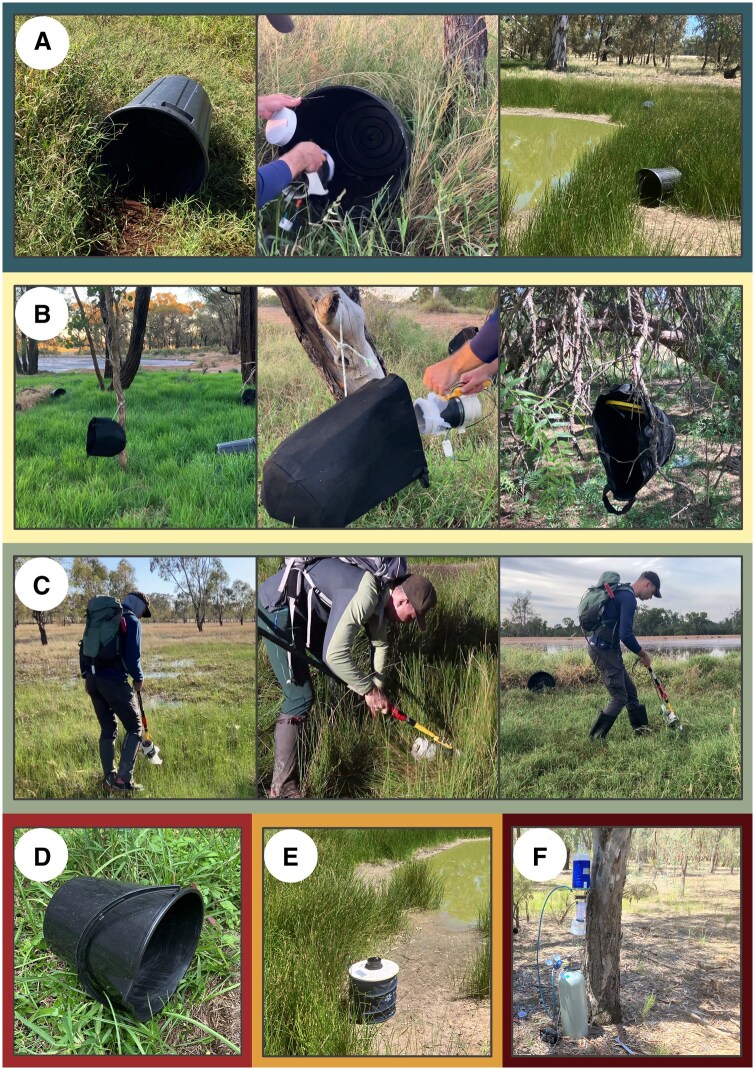
Examples of trap placements and aspiration locations. A) Large bins. B) Felt bags. C) Aspiration. D) Small bin. E) Biogents-Sentinel Trap. F) Pacific Biologics Light Trap.

Large and small bins were placed on their sides within dense ground vegetation, at the base of trees and bushes, or in the open if no ground vegetation was available. Examples of dense ground vegetation included tall green grass, strike rushes, and vegetation in ditches and along the edge of ponds (Fig. 1). Bins were also placed on the edges of the effluent ponds adjacent to the piggeries (where birds roost overnight) to provide a resting spot close to potential bloodmeal sources. The bins were oriented with the opening facing outward from the vegetation and never obstructed. Bins were placed more than 5 m apart along a transect or around the edge of ponds, with the distances between bins primarily determined by variations in the density of vegetation at each sampling location. At some sites, small bins and large bins were placed in different locations, while at other sites they were placed in the same location but set a minimum of 5 m apart to prevent them from directly competing. To prevent the bins from being blown away, rocks were sometimes placed inside. Due to sparse vegetation in many locations, large and small bins could not always be placed in shade. Therefore, collections began 30 min before sunrise the following day and were completed within an hour of sunrise to ensure mosquitoes were collected before wind and temperature increased. Aspirators were used to collect mosquitoes from large bins and small bins.

The felt bags were chosen for their size, dark color, low cost, and ability to be hung from trees. They were made of a black non-woven felt and polyester material. The opening of the felt bags had a diameter of 35 cm, and the bags were 45 cm long. The ends of the felt bags were held open by homemade plastic rings (18 cm diameter) attached to the strings from which the felt bags were hung. Each felt bag cost AUD $2.40. The felt bags were suspended approximately 1 m above the ground, typically from tree branches. In some cases, they were attached to bushes or fallen trees closer to the ground. Proximity to dense ground vegetation was not a criterion for placement but was common. The felt bags were generally spaced about 4 m apart along a transect at the forest edge, intended to offer resting sites for mosquitoes returning to forest cover from more open pasture areas. The felt bags were more widely dispersed than the bins and they were sometimes placed in a different location, depending on the characteristics of the site. Their orientation frequently shifted due to wind-induced spinning. The following day, collections (using the battery powered aspirator) took place at the same time as the other ARS, 30 min before sunrise (Fig. 1).

The BG-S trap is a commercially available mosquito trap that has been demonstrated to occasionally collect bloodfed mosquitoes, although this is not its primary purpose (Roiz et al. 2012, Li et al. 2016, Pruszynski et al. 2020). It was chosen as an example of a readily available trap design that does not require active collection. The trap body is a 40-cm-long cylinder made of a collapsable wire frame and flexible polyester (Fig. 1). In the center of the cover, a small fan sucks air through an intake funnel, which draws mosquitoes into a catch bag. Designed for easy retrieval, the trap’s closing lid activates when the fan shuts off, preventing any mosquitoes from escaping. The fan is powered by a 12 V battery. The BG-S trap can be used with bait and/or CO_2_, but in our comparison, we did not include bait or CO_2_ to decrease cost and increase ease of use. Each BG-S trap cost AUD $309 and each 9 Ah 12 V battery cost AUD $40. The BG-S traps were placed near the large bins, usually within 2 m of the nearest large bin, and with analogous considerations to vegetation type. The fan was turned on in the late afternoon, and the collection bag was retrieved the following day within 1 h of sunrise.

The PB trap is representative of one of the most common methods of mosquito collection, a light trap with supplemental CO_2_ (Silver 2008). Although not designed to collect them, bloodfed mosquitoes are known to enter CO_2_ baited light traps (Vieira et al. 2024). Over the course of a surveillance season, enough bloodfed mosquitoes could reasonably be collected to conduct a vector feeding pattern study (Jansen et al. 2009, Vieira et al. 2024). The PB trap has a 10-cm fan powered by a 6-volt battery with 2 light-emitting diodes (LEDs) above the fan. CO_2_ was provided by a 10 kg (5,000 L) canister and regulator at a rate of approximately 5 L per minute. The PB trap cost $150 AUD, each 12 V 6 Ah battery cost AUD $30, and the CO_2_ per trap night cost approximately AUD $20. PB traps were placed approximately 10 m from the large bins in the same habitat type to allow for a more direct comparison between the 2 collection methods. They were hung at a height of approximately 1 m above the ground from the branch of a tree (Fig. 1). The fan, lights, and CO_2_ were turned on at approximately 5:00 PM, and the collection chamber was collected the following day within 1 h of sunrise.

**Fig. 2.**
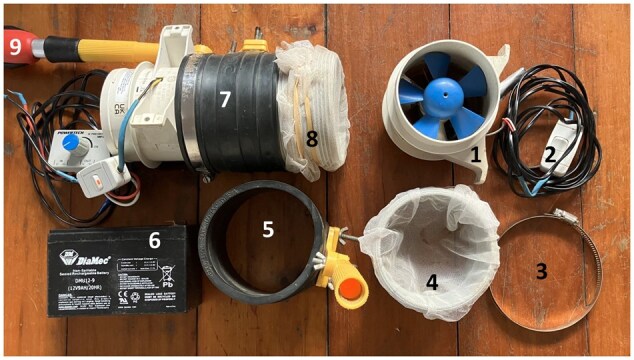
Unassembled and assembled (7) Procopack aspirator with extension pole (9). In-line bilge fan: 90 mm diameter (1). In-line on/off switch (2). Hose clamp (3). Plastic cylinder with mosquito netting over one end; 90 mm diameter (4). Rubber coupler with attachment for pole: 90 mm internal diameter (5). 12 V 9ah lead acid battery (6). Elastic bands to secure mosquito netting (8).

A handheld battery powered aspirator was assembled based on the Prokopack aspirator design detailed in Vazquez-Prokopec et al. 2009. In brief, an in-line bilge fan (90 mm external diameter) was powered by a 12 V battery and connected to a plastic PVC 90 mm diameter cylinder with a length of 100 mm (Fig. 2). A piece of mosquito netting was placed over the end of the plastic cylinder and attached with elastic bands. The fan pulled air through the mosquito netting (and cylinder) at approximately 14 m/s. The aspirator could be attached to a 2-m-long pole to access hard to reach resting locations. The aspirator was used to collect mosquitoes from the large bins, felt bags, and small bins. The aspirator was also used to conduct collections directly from vegetation and other natural features (Fig. 1). Direct aspiration of vegetation took place between sunrise and 3 h after sunrise. The aspirator was used to collect mosquitoes resting in bushes, grass, rushes, tree hollows, bark, and other natural features. Collections were placed in the −60 °C freezer within 30 min and remained frozen until sorting. Of note, the aspirator was used to collect bloodfed mosquitoes from inside the pig sheds, but those collections are not included in this study, as no other method was used in the sheds to provide a comparison point. The materials for the aspirator and battery cost approximately AUD $150.

**Fig. 3.**
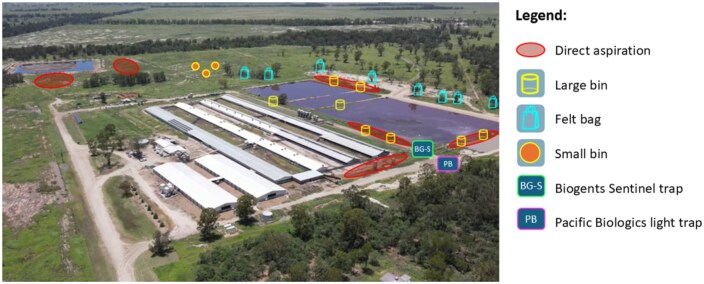
An example of the trap placements and aspiration locations at a commercial piggery in Australia. Icons indicate trap types used at each location but do not reflect the exact number of traps placed.

The trap locations at each piggery were chosen with the goal of understanding the transmission cycle of JEV, particularly the interface between the wild bird and domestic pig populations. Traps were placed next to pig sheds, around the effluent ponds (where birds congregate), and in nearby natural habitats (Fig. 3). Each trap type was placed at multiple locations within each site. ARS were placed in ground vegetation or around the edges of effluent ponds, felt bags were hung in trees or bushes, BG-S and PB traps were positioned near the sheds, and direct aspiration was conducted in areas with dense ground vegetation (Fig. 3). Not all trap types were placed at each location around the piggeries. This was an intentional decision to maximize overall bloodfed mosquito collections at each site by playing to the strengths of each trap type. At all locations with large bins and small bins, aspiration was also conducted. Aspiration was not always conducted at locations with felt bags because there was often no ground vegetation under the trees. The PB and BG-S traps were unavailable at Cattle 1 and Piggery 5 due to limited access to this equipment during the relevant sampling periods (2 d at Cattle 1 and 1 d at Piggery 5).

### Timing the Collection Process

To compare the relative efficiency of bloodfed mosquito collection methods, we measured the total time required to collect mosquitoes using each method. While logistical demands varied (some methods required more setup time or operator involvement), all were influenced by environmental conditions and mosquito density. By quantifying the time investment for each method, we aimed to identify patterns of relative efficiency that reflect both method performance and the constraints of real-world field deployment.

For each method, we recorded the time spent on setup, collection, takedown, and sorting to capture the full effort required to obtain bloodfed mosquitoes. Details of the timing calculations can be found in the code referenced in the data availability statement. Setup time was defined as the time taken to place the traps at each location. The setup time varied between traps because, for example, each felt bag had to be tied to a tree branch, while the PB trap required setup of the CO_2_ canister. For large bins, felt bags, and small bins, the setup time was the sum of all the traps of each type at each location. For example, there would be one setup time recorded for the 10 large bins placed at Site A, Location 1, and a separate setup time recorded for the additional 20 large bins placed at Site A, Location 2. The total time was then divided by the number of bins to give a setup time per bin. The setup times for each PB trap and BG-S trap were recorded separately. Takedown time was defined as the time to retrieve the traps, the opposite of the setup time and with the same considerations. Since traps remained in place for multiple days at each site, setup and takedown times were proportionally distributed in the analysis to allow for fair comparisons. An ARS left in place for several days is inherently more time-efficient than one that requires frequent setup. To standardize comparisons, we assumed a 4-d deployment cycle, meaning the time allocated to a single day of collection included one-quarter of the total setup and takedown time. For methods requiring daily maintenance, such as the CO_2_ trap and Biogents Sentinel trap, additional setup time was included to account for tasks, such as reattaching collection chambers, replacing CO_2_ canisters (CO_2_ trap only), and changing batteries.

Collection time was defined as the time spent retrieving mosquitoes from traps. Again, there was one collection time recorded for all the traps of each type at each location. Each round of direct aspiration had a separate collection time. Usually, we set a timer for 10 min of aspiration, but some aspirations were shorter when the collection chamber filled with mosquitoes or organic matter. It took about 2 min to prepare the aspirator with the battery and mosquito netting. This time was included in the collection time for collections from ARS and the individual aspirations. This is why aspiration does not have a setup time. Finally, sorting time was defined as the time taken to separate bloodfed mosquitoes from unfed females and males. Sorting usually took place at the end of the day. Immediately after collection, mosquitoes were frozen in a −60 °C freezer. At the end of the day, they were spread out on a table and the bloodfed mosquitoes were visually sorted into individual tubes. We chose to record sorting time because the aspirations included lots of bycatch and organic material which increased their sorting times. Comparatively, ARS, PB and BG-S traps collected limited bycatch and essentially no debris, which made sorting bloodfed mosquitoes much easier. Collections from ARS were primarily mosquitoes and were sorted quickly.

To quantify collection efficiency, we calculated the number of bloodfed mosquitoes collected per minute of effort, incorporating all timed activities (setup, collection, takedown, and sorting). At each site, the total time spent on these activities for each method was summed, and the total number of bloodfed mosquitoes collected was divided by the total time invested. For methods deployed at multiple locations within a site, bloodfed mosquito counts and time measurements were calculated separately for each location. Finally, mean efficiencies were calculated by averaging across all sites.

**Table 2.** Collection effort and efficiency of bloodfed mosquito collection methods across sites

	Site							
Collection method	Piggery 1	Piggery 2	Piggery 3	Piggery 4	Cattle 1	Piggery 5	Mean ± SE	**Total** ^a^
**Aspiration**								
Mean setup time per aspiration (minutes)^b^	-	-	-	-	-	-	-	-
Mean collection time per aspiration (minutes)	8.73	7.75	9.81	10.12	11.55	9.00	9.59 ± 0.30	*n* = 103
Mean sorting time per aspiration (minutes)	6.41	3.50	13.24	2.70	8.10	2.10	9.80 ± 0.83	*n* = 103
Total number of aspirations	26	4	56	4	8	3	-	101
Total time all aspirations (minutes)	393.8	45.0	1291.3	51.3	157.0	33.3	-	1,971.7
Total bloodfed mosquitoes—all aspirations	43	1	1719	16	94	2	-	1,875
Efficiency: bloodfed mosquitoes/minute	0.11	0.02	1.33	0.31	0.60	0.06	**-**	0.93
**Large bin**								
Mean setup time per bin (minutes)^c^	1.39	0.67	0.47	0.68	0.42	1.24	0.82 ± 0.15	*n* = 191
Mean collection time per bin (minutes)	0.99	0.78	0.82	0.91	0.81	0.68	0.86 ± 0.05	*n* = 413
Mean sorting time per bin (minutes)	0.41	0.31	0.73	0.43	0.54	0.24	0.47 ± 0.08	*n* = 413
Total time all bins (minutes)	151.6	52.7	186.2	103.4	90.1	43.0	-	627.0
Total number of bins placed at each site	22	21	21	21	20	21	-	126
Total bloodfed mosquitoes per bin	36	5	148	110	55	17	-	371
Efficiency: bloodfed mosquitoes/minute	0.24	0.09	0.79	1.06	0.61	0.40	**-**	0.57
**Felt bag**								
Mean setup time per bag (minutes)	0.88	1.00	0.70	0.79	0.72	0.74	0.79 ± 0.04	*n* = 258
Mean collection time per bag (minutes)	0.53	0.44	0.59	0.66	0.46	0.39	0.54 ± 0.03	*n* = 575
Mean sorting time per bag (minutes)	0.12	0.19	0.25	0.11	0.35	0.08	0.19 ± 0.04	*n* = 575
Total time all bags (minutes)	122.8	90.6	164.3	76.6	87.6	23.0	-	564.9
Total number of bags placed at each site	23	40	56	34	47	19	-	219
Total bloodfed mosquitoes all bags	34	8	41	44	134	0	-	261
Efficiency: bloodfed mosquitoes/minute	0.28	0.09	0.25	0.57	1.53	**-**	**-**	0.43
**Small bin**								
Mean setup time per bin (minutes)	0.85	0.25	0.40	0.67	0.41	0.85	0.63 ± 0.10	*n* = 80
Mean collection time per bin (minutes)	0.63	0.35	0.58	0.59	0.95	0.65	0.14 ± 0.03	*n* = 150
Mean sorting time per bin (minutes)	0.08	0.09	0.11	0.05	0.44	0.18	0.14 ± 0.01	*n* = 150
Total time all bins (minutes)	38.5	11.3	31.5	19.2	31.8	16.8	-	149.1
Total number of small bins at each site	10	10	10	10	10	10	-	60
Total bloodfed mosquitoes all bins	0	0	1	1	9	0	-	11
Efficiency: bloodfed mosquitoes/minute	**-**	**-**	0.03	0.05	0.28	**-**	**-**	0.07
**BG-S trap**								
Mean setup time per trap (minutes)	3.75	4.6	7.0	7.5	-	-	5.35 ± 0.66	*n* = 6
Mean collection time per trap (minutes)	3.85	5.00	2.50	4.50	-	-	3.65 ± 0.32	*n* = 13
Mean sorting time per trap (minutes)	0.83	1.15	1.88	0.50	-	-	1.12 ± 0.38	*n* = 13
Total time all traps (minutes)	26.2	16.9	31.5	17.5	-	-	-	92.1
Total number of traps placed at each site	1	1	1	1	-	-	-	4
Total bloodfed mosquitoes all traps	0	0	8	1	-	-	-	9
Efficiency: bloodfed mosquitoes/minute	**-**	**-**	0.25	0.06	**-**	**-**	**-**	0.09
**PB trap**								
Mean setup time per trap (minutes)	13.15	7.75	12.0	15.0	-	-	12.22 ± 1.01	*n* = 12
Mean collection time per trap (minutes)	5.10	6.12	4.86	7.33	-	-	5.63 ± 0.42	*n* = 24
Mean sorting time per trap (minutes)	2.2	1.58	1.11	2.00	-	-	1.47 ± 0.17	*n* = 24
Total time all traps (minutes)	55.5	46.3	95.8	67.3	-	-	-	264.9
Total number of traps placed at each site	1	2	2	2	-	-	-	7
Total bloodfed mosquitoes traps	0	0	0	3	-	-	-	3
Efficiency: bloodfed mosquitoes/minute	**-**	**-**	**-**	0.04	**-**	**-**	**-**	0.01
**Site bloodfed totals**	113	14	1,908	175	292	19		2,530

a Total number included in the mean. For aspiration, this represents the total number of aspiration events. For all other traps, this corresponds to the total number of individual traps. The total number for setup is lower than that for collection and sorting because traps were set up once but used across multiple days.

b Aspiration does not require a setup.

c Large bins, felt bags, and small bins were not timed individually in the field, but they are presented here individually because the number of traps placed at each location varied across the different sites.

### Mosquito Identification and Bloodmeal Scoring

Bloodfed mosquitoes were identified to the species level by a single trained person using morphological keys (Russell and Debenham 1996). The Sella score is a classification system used to assess the degree of blood meal digestion in mosquitoes, ranging from stages 1 to 7 (Detinova et al. 1962, Santos et al. 2019). Stage 1 represents an empty abdomen, while stage 7 indicates fully developed ovaries. Stages 2 to 6 correspond to varying levels of blood digestion, with stage 2 being a freshly bloodfed mosquito. In this study, bloodfed mosquitoes were assigned a Sella score from 2 to 6 to classify the extent of bloodmeal digestion.

### Model Development

A collection was defined as a single sampling event representing either all the bloodfeds collected in one round of aspiration (typically 10 min) or the total number of bloodfeds captured from a single trap type at a specific location on a given day. If the same trap type was deployed at multiple locations within a site on the same day, each location-trap type combination was considered a separate collection.

To account for variations in the number of ARS deployed at each location, we standardized bloodfed mosquito counts. For large bins, felt bags, and small bins, total bloodfed mosquito counts were divided by the number of bins or bags used and then scaled to a standard; 10 for large and small bins and 20 for felt bags. This was done because we did not always setup the same number of ARS at each location and therefore the setup, collection, and takedown times would not be comparable. Aspiration methods were standardized to a 10-min collection period by adjusting counts proportionally based on the actual collection duration. For the PB trap and BG-S methods, original counts were retained. All standardized counts were rounded to the nearest whole number.

**Fig. 4.**
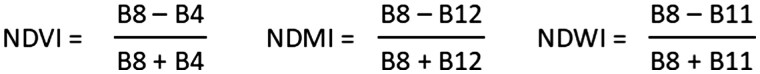
Formulas for the Normalized Difference Vegetation Index (NDVI), Normalized Difference Moisture Index (NDMI), and Normalized Difference Water Index (NDWI), computed using Sentinel-2 bands. The indices are derived from the Red (B4), Near-Infrared (NIR, B8), and Shortwave Infrared (SWIR, B11, and B12) bands.

Environmental conditions influence mosquito resting behavior, movement, and trap success (Silver 2008). To improve model accuracy and ensure key ecological factors were considered, we incorporated climate variables (eg precipitation, temperature) and remotely sensed environmental indices to evaluate their role in trapping success. Daily precipitation data (mm) and daily temperature (°C) were sourced from the Australian Bureau of Meteorology for the nearest weather station to each study site. The weather stations ranged from 2.3 to 15.6 km from the sites. For each collection, cumulative precipitation over the preceding 21 d was calculated. Actual evapotranspiration (ETa) was quantified using the CMRSET Landsat V2.2 dataset, which provides average daily ETa values for each month at 30-m spatial resolution (Guerschman et al. 2022). For each location within each site, mean ETa was calculated over a 30 × 30 m area centered on each location using the value corresponding to the month of sampling. Vegetation and surface water indices were calculated for each location using Sentinel-2 satellite imagery (European Space Agency (ESA) 2015). The Normalized Difference Vegetation Index (NDVI), Normalized Difference Water Index (NDWI), and Normalized Difference Moisture Index (NDMI) were computed as shown in Fig. 4 using Google Earth Engine (Gorelick et al. 2017).

Sentinel-2 imagery was filtered to observations with minimal cloud cover (<15%) during the sampling period, and additional cloud masking was applied using the QA60 band. For each location within each site, a 20-m radius buffer was established to define the area of interest. This buffer was chosen to approximate the spread of ARS at each location. The indices were calculated for each available image within the buffered region, and median values were derived for NDVI, NDWI, and NDMI to represent vegetation greenness, surface water presence, and moisture, respectively. Mean values of the indices within the buffered regions were extracted for each location and used as indicators of environmental conditions during the sampling period.

To evaluate factors influencing bloodfed mosquito collection success, we constructed a generalized linear mixed model with a negative binomial distribution using the glmmTMB package in R version 4.4.1 (Brooks et al. 2017). The final model included standardized bloodfed mosquito counts as the response variable, with fixed effects for collection method, NDWI, temperature, precipitation, and an interaction between collection method and location-level mean count. A nested random effects structure of location/date was used to account for spatial and temporal variation. Model assumptions were assessed using DHARMa (v0.4.7; Hartig et al. 2024), with no substantial overdispersion or residual patterns detected.

Model development proceeded through several stages. We initially evaluated 3 vegetation indices (NDVI, NDMI, and NDWI) separately due to strong collinearity identified via a correlation matrix. NDWI was selected based on superior model performance. Model performance was assessed using Akaike’s Information Criterion (AIC) (Anderson and Burnham 2002). To refine the fixed effects, we applied the dredge function from the MuMIn package (v1.48.11; Bartoń 2025), generating all possible combinations of predictors and ranking them by AIC. For each sampling location, we calculated a location‑level mean count by averaging the collection method standardized counts across all sampling events at that location. This measure reflects relative bloodfed mosquito abundance, rather than a true density estimate, and was used to examine whether collection method performance varied between locations with higher or lower relative abundance. To test for location-level mean count dependent effects, site- and location-level mosquito count variables were calculated and incorporated into models as interaction terms with collection method. AIC comparisons supported the inclusion of the collection method × location-level mean count interaction. The random effects structure was also refined by testing nested and alternative formulations, with the location/date structure offering the best fit. Sensitivity analyses testing for non-linearity, incomplete deployments, and random slopes did not improve model performance.

## Results

A total of 2,530 bloodfed mosquitoes were collected across 6 sites using 6 collection methods. The majority of bloodfed mosquitoes (74%, 1,872/2,530) were collected using aspiration, followed by large bins (15%, 380/2,530), and felt bags (10%, 253/2,530; Table 1). Small bins, BG-S traps, and PB traps collectively contributed less than 1% of the total. Piggery 3 accounted for the largest number of bloodfed mosquitoes (76%, 1,923/2,530), with substantial contributions from Cattle 1 (12%, 304/2,530) and Piggery 4 (7%, 177/2,530). Piggery 1, Piggery 2, and Piggery 5 contributed minimally (combined 5%, 126/2,530).

**Table 1.** Bloodfed mosquitoes collected at each site and with each collection method

	Site						
Collection method	Piggery 1	Piggery 2	Piggery 3	Piggery 4	Cattle 1	Piggery 5	Total
**Aspiration**	**43**	**1**	**1719**	**16**	**94**	**2**	**1875**
*Culex annulirostris*	-	-	1619	11	42	-	1672
*Culex australicus*	1	-	55	4	3	-	63
*Anopheles annulipes*	1	-	8	-	4	-	13
*Aedes theobaldi*	9	-	-	-	-	-	9
*Aedes vittiger*	8	-	-	-	-	-	8
Unidentifiable^a^	24	1	37	1	45	2	110
**Large bin**	**36**	**5**	**148**	**110**	**55**	**17**	**371**
*Cx. annulirostris*	4	4	72	57	11	2	150
*Cx. australicus*	16	-	35	48	-	-	99
*An. annulipes*	6	-	36	2	34	12	90
*Aedes notoscriptus*	-	-	-	1	-	-	1
*Ae. vittiger*	1	-	-	-	-	-	1
Unidentifiable	9	1	5	2	10	3	30
**Felt bag**	**34**	**8**	**41**	**44**	**134**	**0**	**261**
*Cx. annulirostris*	-	-	13	33	-	-	46
*Cx. australicus*	5	7	1	10	-	-	23
*An. annulipes*	26	-	25	-	132	-	183
*Ae. notoscriptus*	-	-	-	1	-	-	1
Unidentifiable	3	1	2	-	2	-	8
**Small bin**	**0**	**0**	**1**	**1**	**9**	**0**	**11**
*Cx. annulirostris*	-	-	1	1	3	-	5
Unidentifiable	-	-	-	-	6	-	6
**BG-S**	**0**	**0**	**8**	**1**	**-**	**-**	**9**
*Cx. annulirostris*	-	-	4	1	-	-	5
*Cx. australicus*	-	-	4	-	-	-	4
**PB LIGHT**	**0**	**0**	**0**	**3**	**-**	**-**	**3**
*Cx. annulirostris*	-	-	-	3	-	-	3
**Site Total**	**113**	**14**	**1,917**	**175**	**292**	**19**	**2,530**

a Bloodfed mosquitoes were sometimes damaged during the collection process, preventing morphological identification. The proportion of unidentifiable specimens differed significantly only between small bins and aspiration, likely reflecting the very low sample size for small bins. When Piggery 3 (which contributed 92% of aspiration-collected mosquitoes) was excluded, large bins and felt bags had a significantly lower proportion of unidentifiable mosquitoes than aspiration based on pairwise 2-proportion z-tests (adjusted *P* < 0.001).

Bold rows are the totals for each collection method and the totals across all collection methods.

*Culex annulirostris* Skuse (Diptera: Culicidae) dominated bloodfed mosquito collections (82%, 2,041/2,530), with smaller collections of *Culex australicus* Dobrotworsky (Diptera: Culicidae) (7%, 191/2,530), *Anopheles annulipes* Walker (Diptera: Culicidae) (5%, 116/2,530), and other species. The proportion of bloodfed *Cx. annulirostris* was highest in aspiration collections (90%, 1,837/2,041). In contrast, felt bags yielded a larger proportion of bloodfed *An. annulipes* (70%, 81/116). A Fisher’s Exact Test with Monte Carlo approximation confirmed a significant association between mosquito species and collection method (*P* = <0.001), indicating that different methods preferentially collect different species.

Efficiency, measured as bloodfed mosquitoes per minute, varied significantly among methods and sites (Table 2). Aspiration was most efficient at Piggery 3 (1.33 bloodfed mosquitoes/minute) and less efficient at other sites. Large bins were most efficient at Piggery 4 (1.06 bloodfed mosquitoes/minute), while felt bags were notably successful at Cattle 1 (1.53 bloodfed mosquitoes/minute). Small bins, BG-S traps, and PB traps exhibited limited efficiency across all sites, contributing minimally to total collections. Bloodfed mosquitoes were most frequently assigned Sella scores of 2 or 3, corresponding to low levels of bloodmeal digestion (Table 3).

**Table 3.** Blood meal digestion stages (Sella scores) of bloodfed mosquitoes collected using different methods

	**Sella score** ^a^				
Collection method	2	3	4	5	6
Aspiration	563 (28%)	630 (31%)	448 (22%)	225 (11%)	149 (7%)
Large bin	181 (46%)	90 (23%)	58 (15%)	42 (11%)	18 (5%)
Felt bag	157 (60%)	56 (21%)	31 (12%)	19 (7%)	0
Small bin	3 (27%)	4 (36%)	2 (18%)	1 (9%)	0
BG-S	6 (66%)	1 (11%)	1 (11%)	1 (11%)	0
PB	0	1 (33%)	2 (67%)	0	0

a A lower Sella score corresponds to a higher blood content and less advanced digestion.

The GLMM indicated that bloodfed mosquito counts varied significantly by collection method, location-level mean count, and environmental conditions (Supplementary Table S1). Felt bags and large bins performed significantly better than direct aspiration at locations with lower mean counts, but their relative performance declined as location-level mean count increased. Small bins were consistently less successful at all sites, both in total count and efficiency. More precipitation in the previous 21 d led to higher bloodfed collections, while higher temperatures led to lower bloodfed collections. Location-level mean count was also positively associated with collection success, underscoring its role in shaping trap performance.

**Fig. 5.**
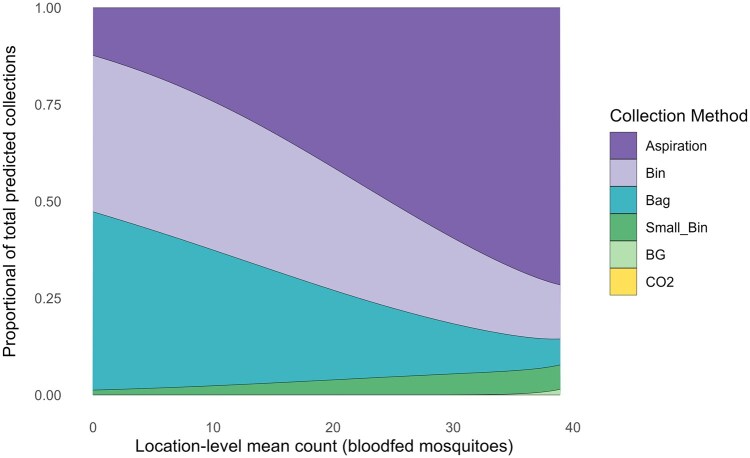
Predicted proportional contributions of collection methods across a gradient of location-level mean counts, derived from a generalized linear mixed model (GLMM) with a negative binomial distribution. The model includes environmental covariates and an interaction between collection method and location-level mean counts. Mosquito count at each location was calculated as the mean standardized mosquito count across all collection events within that location. Abbreviations: PB, Pacific Biologics Light Trap; BG-S, Biogents Sentinel Trap.

The inclusion of the interaction term between collection method and location-level mean count improved model fit compared to simpler models without the interaction (ΔAIC = 34.4), demonstrating that the relationship between collection methods and bloodfed mosquito counts varies meaningfully with bloodfed mosquito mean count across locations (Fig. 5).

## Discussion

**Fig. 6.**
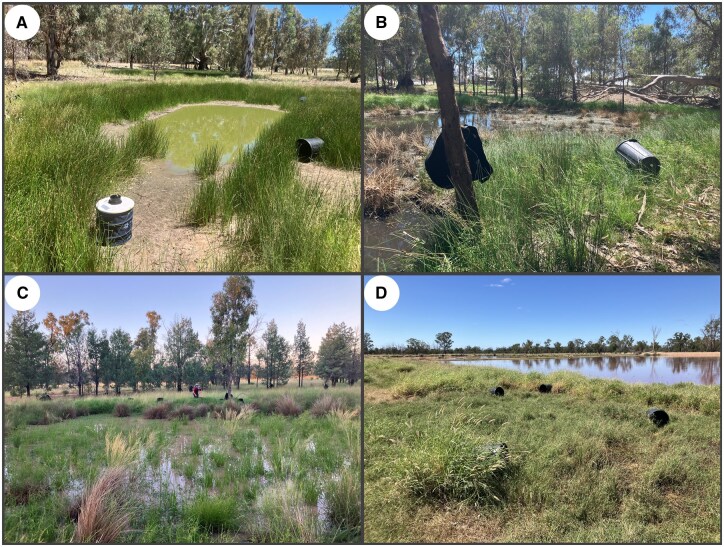
Photos of the 4 locations that yielded the most bloodfed mosquitoes. Panels A and B show Locations N1 and N3 at Piggery 3, respectively; Panel C shows Location 1 at Cattle 1; and Panel D shows Location N1 at Piggery 1.

Despite their importance to understanding vector-borne disease ecology, bloodfed mosquitoes remain difficult to collect in the field. Here, we demonstrate that successful collection of bloodfed mosquitoes depends not only on the collection method but also on selecting a productive location. In locations where resting mosquitoes are abundant, both aspiration and ARS were successful (overall ability to collect bloodfed mosquitoes), with aspiration demonstrating the highest efficiency (number of bloodfed mosquitoes per minute of effort). However, in locations with fewer resting mosquitoes, ARS yielded more consistent returns, underscoring the need to match collection strategies to the local conditions. Placement of ARS within a habitat also influenced the species composition of collections, with ARS hung from tree branches attracting *Anopheles* species and ARS placed on the ground attracting *Culex* species. While aspiration was the most successful method overall, it primarily collected *Culex* species because it was used in ground vegetation, and its success was highly site dependent, driven by exceptionally favorable locations. For example, 2 locations at Piggery 3 (Fig. 6) accounted for 1,502 mosquitoes collected via aspiration (80% of all aspiration collections). These highly productive habitats shared key features: tall ground vegetation, such as grasses, reeds, or rushes growing in extremely dense bunches, and moist soil. Such conditions appear to promote the aggregation of resting *Culex* mosquitoes and support previous findings that mosquitoes preferentially seek out humid, sheltered microclimates to reduce physiological stress and avoid high temperatures (Kirby and Lindsay 2004, Kessler and Guerin 2008). This reinforces the importance of vegetation structure in bloodfed mosquito ecology, echoing earlier work linking shaded or sheltered habitats to improved trapping success (Bidlingmayer 1971, Service 1971, Howard et al. 2011).

Across the varied habitats of this study, ARS proved to be the most consistently successful collection method. Placement of ARS proved critical, both for the total count of bloodfed mosquitoes and species collected. ARS positioned near dense ground vegetation on the edge of standing water consistently yielded more bloodfed mosquitoes than those placed in open locations, likely due to mosquitoes’ preference for sheltered microclimates. These observations align with previous research highlighting the importance of ARS placement (Edman et al. 1968) and design features such as box size and unobstructed openings (Nelson and Spadoni 1972, Reisen et al. 1983, Meyer 1985, Burkett-Cadena et al. 2008). Interestingly, felt bags were also more successful when placed near dense ground vegetation, even though they were hung 1 m above the ground and primarily attracted arboreal species such as *Anopheles annulipes* Walker.

Distinct resting site and trap choice patterns were observed among species in our collection. *An. annulipes* showed a pattern of resting in elevated sites, consistent with findings by Kay (1983), who collected this species mainly from tree trunks and tree holes in Queensland. In our study, felt bags placed about 1 m above the ground collected a significantly more *An. annulipes* than either nearby large bins or aspiration, both in absolute and relative terms. Ground-level methods, including large bins and aspiration, primarily captured *Culex* species. Likewise, Kay (1983) noted that *Cxulex annulirostris* Skuse could be collected in Australia from most shaded, moderately dense herbage but that abundance varied between locations and seasons, with *Cx. annulirostris* sometimes resting in trees when ground cover was sparse. This spatial differentiation between species in resting behavior has been observed in other countries and highlights the importance of considering vertical stratification when designing collection methods, particularly for studies targeting specific species (Sauer et al. 2021). Given that *Cx. annulirostris* is the primary Australian vector of JEV and is frequently associated with dense ground vegetation, surveillance, and residual spraying efforts may be more effective when focused on these locations (Kay 1983, van den Hurk et al. 2022).

ARS were more efficient than aspiration at locations with fewer bloodfed mosquitoes (Fig. 5). Several factors may have contributed to this difference. If the species most commonly collected across all sites prefer to rest in cavities (particularly *An. annulipes*), then ARS may attract these species and congregate them into a convenient location for collection. This effect may be less noticeable for non-cavity resting taxa, such as *Aedes* (Burkett-Cadena et al. 2019); however, our sample size for this genus is too small to draw a conclusion. Aspirating from vegetation is difficult because resting mosquitoes can disperse more widely, escape in any direction, and the aspirator’s airspeed (approximately 14 m/s) extends only a few centimeters beyond the collection chamber entrance, requiring the operator to bring the aspirator very close to the resting mosquitoes. In contrast, ARS have a single opening, limiting escape routes and directing mosquitoes into the aspirator as they fly out. Because the Prokopack aspirator has a smaller opening than the large bins and felt bags, some mosquitoes escaped capture even with careful technique, such as holding the aspirator just outside and near the top of the opening, tapping the ARS to prompt flight, and not accidentally blowing the mosquitoes away with the fan. Other ARS designs such as the pop-up resting shelter by Burkett-Cadena et al. (2019) can greatly improve retention. Additionally, ARS reduce post-­collection processing time as they do not accumulate organic material and bycatch. Friesen and Johnson (2013) came to a similar conclusion when they demonstrated that fiber pots were a more efficient method for bloodfed mosquito collection than direct aspiration, particularly when sorting time was factored in. BG-S traps were not efficient at collecting bloodfed mosquitoes, even though their design prevents mosquitoes escaping. This finding supports previous research that demonstrated that BG-S traps are most effective at collecting host-seeking *Aedes* mosquitoes and that aspiration collects more bloodfeds than BG-S (Williams et al. 2006).

Our model identified significant associations between bloodfed mosquito collections, temperature (positively associated), and precipitation (negatively associated); however, these findings should be interpreted with caution given the study’s observational design. All but one site were commercial piggeries with broadly similar environments, and because sampling was undertaken over consecutive days at each site, and temperature and precipitation varied minimally across collection days, these results may reflect site-level differences rather than direct causal relationships. The one site with a notably different landscape and climate was Piggery 5 in the Atherton Tablelands area near Cairns in Far North Queensland, a humid tropical region with heavy summer rainfall. The pastures and collection locations around Piggery 5 were waterlogged with recent rainfall. Collection efficiencies were low for all methods, with large bins being the only successful method, potentially reflecting the difficulty of collecting bloodfed mosquitoes in climates with high precipitation that provide extensive resting habitat (Burkett-Cadena et al. 2019). Interestingly, *An. annulipes* were collected from the large bins placed within dense grass rather than the felt bags hung in nearby trees, which had dry ground beneath them. In contrast, Piggery 2, which had the driest habitat and least precipitation, also yielded few bloodfed mosquitoes, possibly due to a lack of larval habitat or humid resting locations. The few bloodfed mosquitoes we collected from Piggery 2 came from one small grassy area with standing water right next to the pig shed and from one very dense deciduous tree in a dry field. Future surveys could be strengthened by incorporating repeated sampling across a broader range of environmental conditions, especially in tropical habitats with known JEV transmission. Including systematic assessments of larval and adult mosquito habitats would also help clarify how environmental features shape resting behavior.

To maximize the success of bloodfed mosquito collections, collection strategies should prioritize initial habitat assessment. When targeting species such as *Cx. annulirostris*, operators should search the area for dense ground vegetation near small bodies of water or with humid soil, then manually part the vegetation and visually inspect for resting mosquitoes. If the vegetation contains a large number of visible resting mosquitoes, aspiration should be prioritized; however, if resting mosquitoes are not present in large abundance, surveillance should prioritize placing ARS within dense ground vegetation. Likewise, when targeting species that rest in tree cavities, such as *An. annulipes*, locate a dense-canopied tree ideally adjacent to small pools of standing water, dense ground vegetation and place ARS within the canopy for optimal efficiency. It is crucial to collect from ARS at dawn before the temperature and wind increase. Based on the initial collections, modify the location of the ARS and aspiration to increase collections of target species.

Bloodfed mosquitoes are challenging to collect, yet they are key to understanding how pathogens JEV are transmitted between species. This study evaluated different methods for collecting bloodfed mosquitoes, demonstrating that ARS are the most successful method in locations with fewer bloodfed mosquitoes. Direct aspiration was the most successful and efficient method in locations with high bloodfed mosquito numbers and useful for short-term collections of bloodfed mosquitoes in remote locations. PB light traps, BG-S traps and small bins were not successful methods. By identifying where each method performs best, this study will streamline future collections. These insights support more strategic, efficient, and scalable approaches to mosquito surveillance and are informative to further research using bloodfed mosquitoes to improve our understanding of mosquito-borne disease ecology, transmission, and risk management.

## Supplementary Material

tjaf139_Supplementary_Data

## Data Availability

The code underlying this article are available in https://github.com/ThaboMoore/Bloodfed-Collections.
