# Supplementary material for: Assessing the performance of bloodfed mosquito collection strategies in Australia
Source: J Med Entomol. 2025 Oct 16;63(1):tjaf139. doi: 10.1093/jme/tjaf139 (PMC12823276; doi:10.1093/jme/tjaf139)
Supplement: tjaf139_Supplementary_Data [file tjaf139_supplementary_data.docx]

| **Supplementary table 1.** Fixed and random effect estimates from the negative binomial GLMM on bloodfed mosquito collections | | | | | | |
| --- | --- | --- | --- | --- | --- | --- |
| **Category** | **Term** | **Estimate** | **Standard error** | **95% CI lower** | **95% CI upper** | **p-value** |
| Fixed Effect | Aspiration (Intercept) | -0.40 | 1.28 | -2.91 | 2.10 | 0.75 |
| Fixed Effect | Felt bag | 1.32 | 0.45 | 0.44 | 2.20 | 0.00 |
| Fixed Effect | BG-S | -21.05 | 15756.40 | -30903.02 | 30860.93 | 1.00* |
| Fixed Effect | Large bin | 1.19 | 0.38 | 0.44 | 1.93 | 0.00 |
| Fixed Effect | PB trap | -17.94 | 5235.42 | -10279.17 | 10243.29 | 1.00 |
| Fixed Effect | Small bin | -2.28 | 1.03 | -4.30 | -0.26 | 0.03 |
| Fixed Effect | Location-density | 0.12 | 0.02 | 0.08 | 0.15 | 0.00 |
| Fixed Effect | NDWI | 0.77 | 2.81 | -4.73 | 6.27 | 0.78 |
| Fixed Effect | Precipitation | -0.08 | 0.02 | -0.12 | -0.04 | 0.00 |
| Fixed Effect | Temperature | 0.15 | 0.05 | 0.06 | 0.24 | 0.00 |
| Fixed Effect | Felt bag:Location-density† | -0.09 | 0.02 | -0.13 | -0.06 | 0.00 |
| Fixed Effect | BG-S:Location-density | 0.44 | 404.94 | -793.23 | 794.11 | 1.00 |
| Fixed Effect | Large bin:Location-density | -0.07 | 0.02 | -0.11 | -0.04 | 0.00 |
| Fixed Effect | PB trap:Location-density | -4.31 | 58260.46 | -114192.72 | 114184.10 | 1.00 |
| Fixed Effect | Small bin:Location-density | 0.00 | 0.07 | -0.14 | 0.14 | 0.96 |
| Random Effect | Date:Location‡ | 0.22 |  |  |  |  |
| Random Effect | Location | 0.36 |  |  |  |  |
| Model Fit Statistic | AIC | 1002.87 |  |  |  |  |
| Model Fit Statistic | Dispersion Parameter | 1.33 |  |  |  |  |
| Model Fit Statistic | Log-Likelihood | -483.44 |  |  |  |  |
| Abbreviations: AIC, Akaike Information Criterion; CI, Confidence Interval; GLMM, Generalized Linear Mixed Model; NDWI, Normalized Difference Water Index; PB, Pacific Biologics Light Trap; BG-S, Biogents Sentinel Trap.  * BG-S and PB traps exhibited extremely low bloodfed mosquito collections, resulting in large standard errors and wide confidence intervals. Consequently, their estimated effects are highly uncertain, and the lack of statistical significance (p = 1.00) should be interpreted with caution.  † Interaction terms (e.g., Method:Location-density) represent how the effect of each collection method varies with local mosquito density.  ‡ Random effects were included to account for spatial (Location) and temporal (Date:Location) variability in mosquito collections. | | | | | | |

| **Supplementary table 2.** Characteristics of sampling sites | | | | | | | |
| --- | --- | --- | --- | --- | --- | --- | --- |
| **Site** | **Location** | **Latitude** | **Days** | **Month, Year** | **Piggery size*** | **Median cumulative summer precipitation (Nov–Mar)** | **Mean daily maximum temperature**  **(Nov-Mar)** |
| Piggery 1 | Southern Queensland | -28.3 | 5 | November, 2023; March, 2024 | 6 sheds; 3.42 ha of sheds | 314.0 mm  (IQR: 250.6–389.5) | 32.23 ± 1.07 °C |
| Piggery 2 | Northern Victoria | -36.4 | 2 | February, 2024 | 5 sheds; 0.99 ha of sheds | 173.2 mm  (IQR: 119.4–205.8) | 29.81 ± 0.74 °C |
| Piggery 3 | Northern Victoria | -36.7 | 5 | February, 2025 | 5 sheds; 0.76 ha of sheds | 178.4 mm  (IQR: 123.3–239.8) | 27.72 ± 1.36 °C |
| Piggery 4 | Northern Victoria | -36.8 | 2 | February, 2025 | 9 sheds; 1.96 ha of sheds | 163.4 mm  (IQR: 120.4–208.2) | 27.51 ± 1.22 °C |
| Cattle 1 | Southern Queensland | -27.9 | 2 | 08.03.24 – 09.03.24 | NA | 364.1 mm  (IQR: 294.5–457.8) | 32.23 ± 1.07 °C |
| Piggery 5 | Far north Queensland | -17.4 | 2 | 26.03.24 – 27.03.24 | 1 shed; 0.1 ha of sheds | 805.3 mm  (IQR: 649.9–1071.3) | 29.25 ± 1.08 °C |
| *While we did not have access to precise pig numbers at each site, industry data suggest that commercial piggery sheds in Australia typically house between 200 and 1,000 pigs, depending on pig age, housing system, and stocking density (Australian Pork Limited 2023). We therefore provide shed area (in hectares) as a proxy for relative piggery size. | | | | | | | |
